# Preoperative mapping techniques for brain tumor surgery: a systematic review

**DOI:** 10.3389/fonc.2024.1481430

**Published:** 2025-01-07

**Authors:** Augusto Leone, Francesco Carbone, Uwe Spetzger, Peter Vajkoczy, Giovanni Raffa, Flavio Angileri, Antonino Germanó, Melina Engelhardt, Thomas Picht, Antonio Colamaria, Tizian Rosenstock

**Affiliations:** ^1^ Department of Neurosurgery, Charité – Universitätsmedizin Berlin, Corporate Member of Freie Universität Berlin, Humboldt-Universität zu Berlin, and Berlin Institute of Health, Berlin, Germany; ^2^ Department of Neurosurgery, Städtisches Klinikum Karlsruhe, Karlsruhe, Germany; ^3^ Department of Neurosurgery, University of Foggia, Foggia, Italy; ^4^ Department of Neurosurgery, University of Messina, Messina, Italy; ^5^ Cluster of Excellence: “Matters of Activity. Image Space Material,” Humboldt University, Berlin, Germany; ^6^ Berlin Institute of Health at Charité – Universitätsmedizin Berlin, Berlin Institute of Health (BIH) Biomedical Innovation Academy, BIH Charité Digital Clinician Scientist Program, Berlin, Germany

**Keywords:** navigated transcranial magnetic stimulation (nTMS), magnetoencephalography (MEG), fMRI, brain mapping, preoperative mapping, brain tumor surgery, motor eloquent tumors, language eloquent tumor

## Abstract

Accurate preoperative mapping is crucial for maximizing tumor removal while minimizing damage to critical brain functions during brain tumor surgery. Navigated transcranial magnetic stimulation (nTMS), magnetoencephalography (MEG), and functional magnetic resonance imaging (fMRI) are established methods for assessing motor and language function. Following PRISMA guidelines, this systematic review analyzes the reliability, clinical utility, and accessibility of these techniques. A total of 128 studies (48 nTMS, 56 fMRI, 24 MEG) were identified from various databases. The analysis finds nTMS to be a safe, standardized method with high accuracy compared to direct cortical stimulation for preoperative motor mapping. Combining nTMS with tractography allows for preoperative assessment of short-term and long-term motor deficits, which may not be possible with fMRI. fMRI data interpretation requires careful consideration of co-activated, non-essential areas (potentially leading to false positives) and situations where neural activity and blood flow are uncoupled (potentially leading to false negatives). These limitations restrict fMRI’s role in preoperative planning for both motor and language functions. While MEG offers high accuracy in motor mapping, its high cost and technical complexity contribute to the limited number of available studies. Studies comparing preoperative language mapping techniques with direct cortical stimulation show significant variability across all methods, highlighting the need for larger, multicenter studies for validation. Repetitive nTMS speech mapping offers valuable negative predictive value, allowing clinicians to evaluate whether a patient should undergo awake or asleep surgery. Language function monitoring heavily relies on the specific expertise and experience available at each center, making it challenging to establish general recommendations.

## 1 Introduction

Gliomas represent the most common intracranial malignant pathology in pediatric and adult populations (1). The extent of resection (EOR) represents an independent prognostic factor predicting the survival and quality of life of these patients (2–6). Therefore, preoperative planning as well as intraoperative mapping of cortical and subcortical hotspots is of utmost importance in neuro-oncological surgery (7, 8).

Intraoperative direct electrical stimulation (DES) is considered the gold standard for creating a map of the functional areas within and around the lesion and is commonly used to map language and sensorimotor function (9–11). The suitability for an awake surgery depends on various factors such as the patient’s personality and preoperative general/neurological condition, so not every patient is eligible for an awake surgery.

In recent years, significant advancements have been made in the field of preoperative functional mapping, with numerous international experts exploring the clinical applications of instruments such as navigated transcranial magnetic stimulation (nTMS), functional magnetic resonance imaging (fMRI), and magnetoencephalography (MEG) (12–14). The objective of this systematic review is to comprehensively and critically assess the role, advantages, and limitations of nTMS, fMRI, and MEG in preoperative mapping for managing patients with motor- and language-eloquent gliomas, and how these techniques may impact the extent of resection (EOR) and functional outcomes. In detail, the accuracy, resolution, reliability, and accessibility were investigated. Furthermore, new insights into the procedural intricacies of these methods are provided to enhance understanding of the current non-invasive solutions for sensorimotor and language mapping.

## 2 Materials and methods

### 2.1 Search strategy and selection criteria

**Figure 1:**
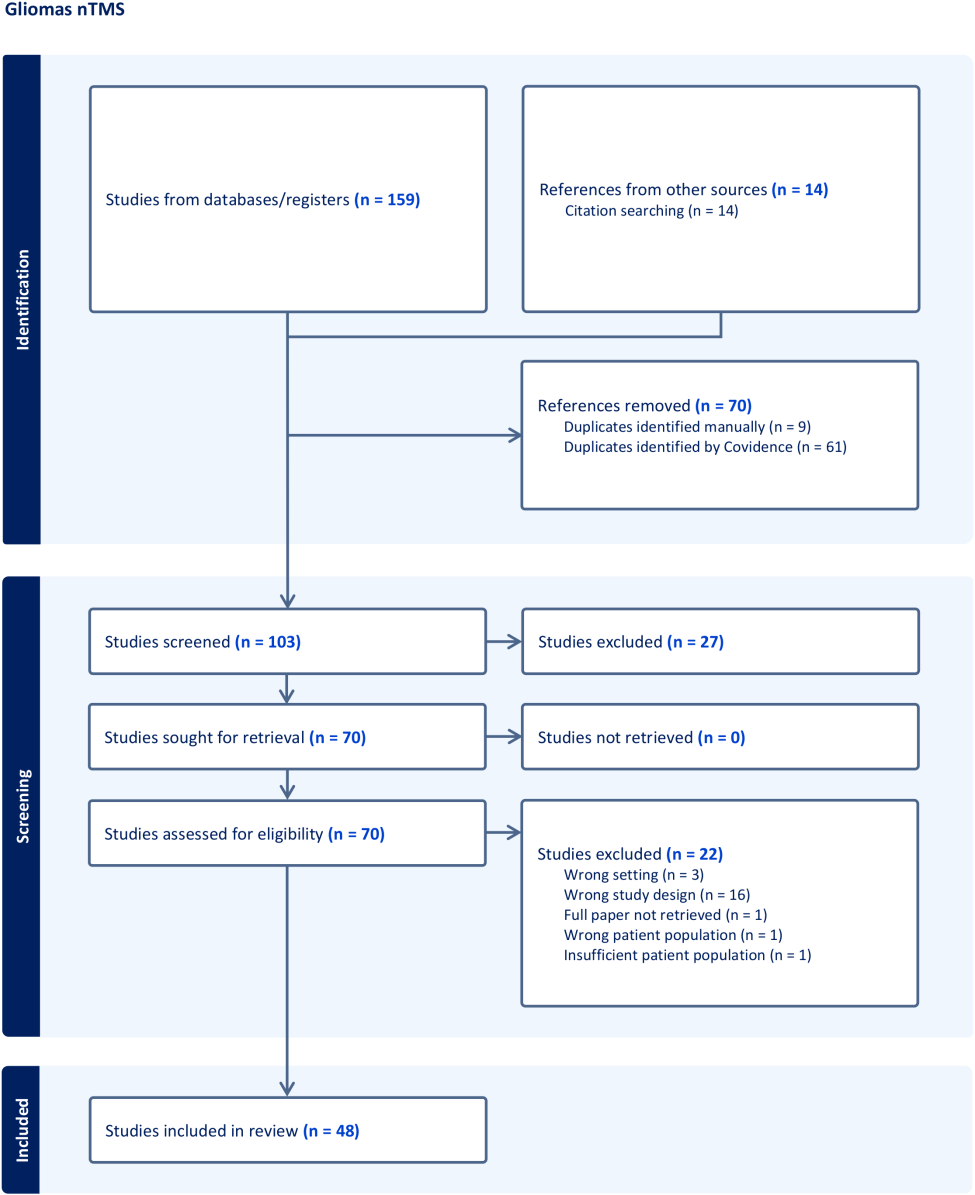
Workflow of the systematic research concerning nTMS following PRISMA guidelines.

A comprehensive, systematic search of the literature was performed in compliance with the updated Preferred Reporting Items for Systematic Reviews and Meta-Analyses (PRISMA) 2020 guidelines as shown in **Figure 1**. The literature review for articles was conducted in January 2023 using electronic databases including MEDLINE/PubMed, EMBASE, PLOS, and the Cochrane Library. Human studies published in English between 1997 and January 2024 were considered for inclusion. The primary search terms used were “fMRI”, “functional Magnetic Resonance Imaging”, “nTMS”, “[navigated] Transcranial Magnetic Stimulation”, “MEG”, “Magnetoencephalography”, “MSI”, and “magnetic source imaging”, “glioma”, and “preoperative mapping” in the titles and abstracts of articles using various MeSH combinations. Eligible articles consisted of original studies and experiences involving cohorts of more than 10 patients. Exclusion criteria were applied to case reports, publications detailing technical notes, and studies where gliomas made up less than 20% of the overall cohort.

### 2.2 Data extraction

Two authors independently reviewed all abstracts to recognize articles that warranted a full-text review. The abstracts were assessed against predetermined eligibility criteria, and all included studies were reviewed with a third author. The following information was obtained: the author’s name, country, publication year, number of patients, type of lesion, tumor localization, histology, and type of preoperative mapping. In particular, details on accuracy, resolution, reliability, accessibility, and patient comfort techniques were extracted for all three mapping techniques. The gathered data were stored in a centralized database using Microsoft Excel. We evaluated the methodological quality of the studies (to assess bias risk) using the JBI Critical Appraisal Checklist for Case Series. This system assigns a quality rating to case series ranging from 0 (poor methodological quality) to 10 (optimal methodological quality) (15, 16).

## 3 Results

**Figure 2:**
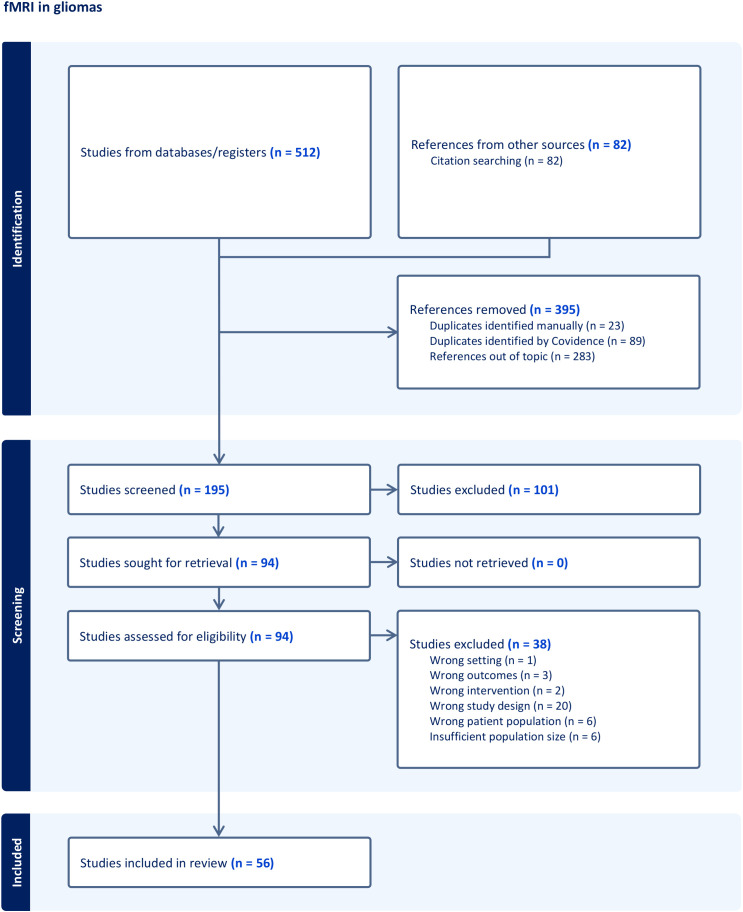
Workflow of the systematic research concerning fMRI following PRISMA guidelines.

**Figure 3:**
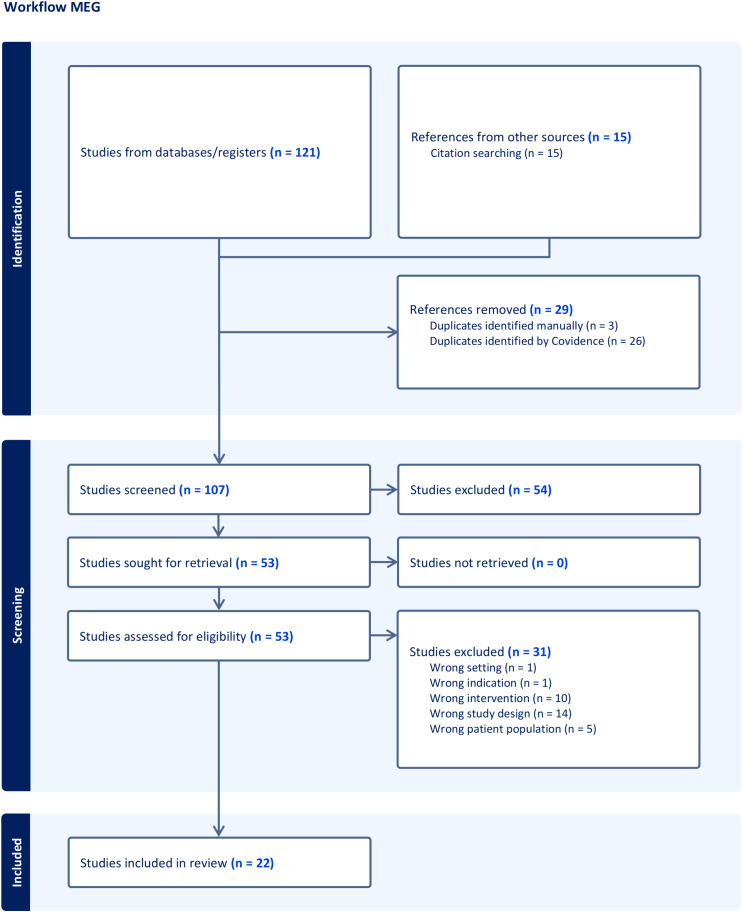
Workflow of the systematic research concerning MEG following PRISMA guidelines.

A total of 907 records were identified and were then subdivided into three groups according to the described technique: nTMS (**Figure 1**), fMRI (**Figure 2**), and MEG (**Figure 3**). Any irrelevant research, review articles, meeting abstracts/summaries, editorials, and studies lacking data on post-operative neurological outcomes were excluded. Thus, 229 full texts were assessed for eligibility, with 99 studies being excluded for various reasons: inappropriate study design (n = 50), inappropriate setting (n = 5), inappropriate patient population (n = 14), insufficient population size (n = 7), inappropriate intervention (n = 12), inappropriate outcomes (n = 3), through an automated system (Covidence) and one paper was not retrieved. Finally, 128 publications were included in the qualitative analysis: 48 nTMS studies (**Table 1**), 56 fMRI studies (**Table 2**), and 24 MEG studies (**Table 3**).

**Table 1:** Extraction of the most significant studies investigating nTMS's role as a preoperative mapping tool.

Author	Year	Country	Aim of the study	N. of patients	Mean age (Range)	N. of gliomas	Results
**Picht T.**	2013	DE	To compare the safety and effectiveness of preoperative nTMS with DCS mapping during awake surgery for the identification of language areas in patients with left-sided cerebral lesions.	20	48 (-)	18	nTMS maps showed an overall sensitivity of 90.2%, specificity of 23.8%, positive predictive value of 35.6%, and negative predictive value of 83.9% compared with DCS. For the anatomic Broca’s area, the corresponding values were a sensitivity of 100%, specificity of 13.0%, PPV of 56.5%, and NPV of 100%, respectively.
**Conti A.**	2014	ITA	To investigate the effectiveness of nTMS-based DTI tractography compared to anatomical DTI tractography	20	51,4 (19-76)	14	nTMS-DTI reconstructed a decreased number of fibers and a greater overlap of the motor cortex and the cortical end-region of the CST
**Frey D.**	2014	DE	To investigate the impact of nTMS on outcomes compared to historical control group without nTMS	250	54 (19-82)	128	In comparison to the control group, nTMS patients had a higher rate of GTR, a better PFS, and fewer postoperative deficits (although not statistically significant). nTMS led to more extensive resection in 35.2% of cases and expanded surgical indication in 14.8%. 3.5% of patients had a more limited resection due to nTMS findings
**Krieg S.**	2014	DE	To investigate the impact of nTMS on outcomes in motor-eloquent tumors compared to historical control group without nTMS	100	53,1 (-)	66	Patients in the nTMS group showed a significantly lower rate of residual tumor on postoperative MRI (OR 0.3828; 95% CI 0.2062–0.7107). Rate of permanent motor worsening: 13% nTMS and 18% non-nTMS group (p = .0057).
**Picht T.**	2015	DE	To identify the additive impact of presurgical nTMS for motor-eloquent lesions compared to patients treated without preoperative nTMS	127	53,7 (20-79)	127	Lower residual tumor volume in nTMS group (5.9ml vs. 9.6ml) (p < 0.05). Shorter surgery time in nTMS group (mean time saving: 9.6 %)
**Krieg S.**	2015	DE	To compare the clinical course of patients with motor eloquently located supratentorial HGG who underwent preoperative nTMS with a historic control group of patients who were operated on without nTMS data by a matched pair analysis.	140	59,2 (-)	140	Lower rate of STR in nTMS group (34.3% vs 54.3%; p = 0.0172). Lower rate of unexpected tumor residuals in nTMS group (15.7% vs 32.9%; p = 0.0180). 60.0% of patients of the nTMS group and 54.3% of patients of the non-nTMS group were eligible for postoperative chemotherapy (OR 1.2630, CI 0.6452 – 2.4710; p = 0-4945), while 67.1% of nTMS patients and 48.6% of non-nTMS patients received radiotherapy (OR 2.1640, CI 1.0910 – 4.2910; p = 0.0261). Moreover, 3-, 6- and 9-months survivals was significantly better in the nTMS group (p = 0.0298, p = 0.0015 and p = 0.0167). Significantly smaller craniotomies in nTMS group (25.3 ± 9.7 cm^2^ vs 30.8 ± 13.2 cm^2^; p = 0.0058)
**Raffa G.**	2017	ITA	To evaluate the impact of nTMS and nTMS-based DTI tractography on the functional outcome	16	50,2 (28-71)	16	In 12 patients (75%), nTMS added useful information on functional anatomy and surgical risks. Surgical strategy was modified in 9 of 16 cases (56%). Preoperative nTMS mapping + nTMS-based tractography provided a good outcome at discharge, with a decrease in postoperative motor and/or language deficits, as compared with controls (6 vs. 44 %; p = 0.03).
**Butenschön V.M.**	2018	DE	To analyze the cost-effectiveness of nTMS	1000	58 (-)	1000	The mean additional cost for nTMS was 7969 euros, which corresponded to a mean increase in QALY of 0.18 (45086/QALY, meaning cost-effectiveness according to WHO CHOICE guidelines – Threshold < 144570 euros for Germany)
**Jung J.**	2018	UK	Impact of nTMS motor and language mapping on surgical planning	35	47 (19-70)	31	nTMS resulted in change of the surgical strategy in 31.5% (craniotomy size 7; access pathway 3; surgical indication 1). Very high agreement between nTMS and DCS hotspot (mean abductor pollicis brevis hotspot distance of 3.50 +- 0.66 mm). The specificity of nTMS for language was 66.7%, with a negative predictive value of 74.1%.
**Raffa G.**	2018	ITA	To analyze the impact of nTMS-based tractography for motor-eloquent lesions compared with historical control group	105	-	75	The patients in group A (just nTMS) and B (nTMS + nTMS-based tractography) received smaller craniotomies (p = .01; p = .001), had less postoperative seizures (p = .02), a better postoperative motor performance (p = .04) and higher Karnofsky Performance Status (p = .009) than group C (no nTMS nor tractography)
**Sollmann N.**	2019	DE	Measuring lesion-to-tract distances (LTD) using nTMS-based language tractography to predict postoperative language outcome	50	-	-	LTDs of ≥ 8 mm (AF) and ≥ 11 mm (SLF, ILF, UC, or IFOF) were determined as cut-off values for surgery-related permanent aphasia
**Sollmann N.**	2020	DE	Measuring lesion-to-tract distances (LTD) using nTMS-based motor + language tractography to predict postoperative motor + language outcome	216	57 (19-89)	189	The cut-off values for surgery-related paresis were ≤12 mm (LTD—CST) and for aphasia ≤16 mm (LTD—AF) or ≤25 mm (LTD—another closest language-related tract), respectively.
**Belotti F.**	2021	DE	Association between nTMS-based motor tractography, extent of resection and motor outcome	183	50 (21-81)	183	TTD (Tumor-tract distance) correlates with the EOR. TRD (Tumor-resection cavity distance) showed a good correlation with longterm motor outcome, with no new permanent deficits if TRD > 8 mm.
**Hendrix P.**	2021	DE	Impact of nTMS motor-mapping on EOR and motor outcome – matched pair analysis (historical control group)	105	62,5 (-)	52	GTR was more frequently achieved in the nTMS group compared to the non-nTMS group: 81.9% vs 69.5% (p = .024). Motor outcome did not differ (P = .344)
**Ille S.**	2021	DE	Nonrandomized comparison of awake vs. asleep surgery in patients who underwent rTMS language mapping + tractography	147	54 (20-84)		The functional outcome did not differ between groups. GTR was achieved in more cases in the asleep group (87%, vs. 72% in the awake group, p = 0.04)
**Rosenstock T.**	2021	DE	To analyze to what extent preoperative nTMS motor risk stratification can improve the interpretation of IOM phenomena	66	48 (-)	66	Motor outcome of irreversible MEP amplitude decreases ≤50% depending on nTMS-based risk stratification. Risk of new postoperative paresis at subcortical stimulation intensities ≤5mA moderate to high (depending on nTMS-based risk stratification)
**Rosenstock T.**	2021	DE	To validate the nTMS-based risk stratification model for the prediction of new postoperative motor deficits	165	50 (-)	165	Infiltration of primary motor cortex, TTD < 8 mm and a FA value of the corticospinal tract < 0.47 were confirmed as risk factors for the development of new postoperative motor deficit.

PPV, positive predictive value; NPV, negative predictive value; PFS, progression free survival; LTD, lesion-to-tract distance; AF, arcuate fasciculus; FA, fractional anisotropy; ILF, inferior longitudinal fascicle; UF, uncinate fascicle; FoF, fronto occipital fascicle; TTD, tumor-to-corticospinal tract distance; TRD, corticospinal tract-to-resection cavity distance; EOR, extent of resection; MEG, Magnetoencephalography; nTMS, navigated transcranial magnetic stimulation; fMRI, functional magnetic resonance imaging.

**Table 2:** Extraction of the most significant studies investigating fMRI’s role as a preoperative mapping tool.

Author	Year	Country	Aim of study	N. of patients	Mean age (Range)	N. of gliomas	Results
**Gumprecht H.**	2002	DE	To assess the role of preoperative fMRI mapping combined with intraoperative neuronavigation	15	–	11	There was no neurological deterioration postoperatively, 12 patients remained unchanged, one patient improved from his hemiparesis and one patient had no more seizure postoperatively. MRI acquired within 24 hours postoperatively demonstrated complete tumor removal in 7 cases and residual tumors in 8 cases
**Stippich C.**	2007	DE	To prospectively identify Broca’s and Wernicke’s areas with standardized presurgical fMRI	81	42 (7-75)	58	Success rates in localizing Broca’s and Wernicke’s areas were 96% with the SG paradigm, 81% and 80% with the WG paradigm for Broca’s and Wernicke’s areas, respectively, and 98% for both areas when the SG and WG paradigms were combined
**Picht T.**	2008	DE	To investigate the concordance between fMRI and DCS	30	56,7 (33-80)	24	The distance between the fMRI and the DCS “hot spots” was on average 13.8 mm. (range: 7–28 mm). The fMRI “hot spots” lay predominantly medially from the DES “hot spots”
**Peck K.K.**	2009	USA	To investigate the ability of fMRI to measure language dominance in previously operated patients	26	65,4 (35-71)	26	In patients with previous surgery, the concordance with intraoperative findings was 75% for Broca’s area and 88% using hemispheric ROIs
**Forster M.T.**	2011	DE	To evaluate the reliability of nTMS compared with fMRI and DCS for preoperative planning	10	41,9 (20-63)	10	Distances from nTMS to DCS (10.5 +/- 5.67 mm) were significantly smaller than those from fMRI to DCS (15.0 +/- 7.6 mm)
**Coburger J.**	2013	DE	To evaluate the advantage of nTMS in comparison with fMRI for preoperative mapping of the Rolandic region	30	47,8 (2-76)	15	The mean accuracy of nTMS was higher than fMRI. In the subgroup of intrinsic tumors, nTMS produced significantly higher accuracy scores in the mapping of the lower extremity. fMRI failed to localize hand or leg areas in 6 out of 23 cases
**Trinh V.T.**	2014	USA	To study the role of fMRI in preventing neurological injury in awake craniotomy patients	214	44 (18-74)	214	In 40% of our cases (n = 85) fMRI was utilized for the intraoperative localization of the eloquent cortex. In the other 129 cases significant noise distortion, poor task performance and nonspecific BOLD activation precluded the surgeon from using the fMRI data. Compared with DCS, fMRI had a sensitivity and specificity, respectively, of 91 and 64% in Broca’s area, 93 and 18% in Wernicke’s area and 100 and 100% in motor areas
**Bailey P.D.**	2015	USA	To determine whether LAD calculated on presurgical BOLD fMRI and degree of white matter involvement predict perioperative motor and language deficits	76	47,4 (15-78)	70	In symptomatic patients, motor and expressive language LAD were significantly lower (z = –3.78, P = .0002, and z = –2.51, P = .01, respectively) than in asymptomatic patients. The degree of CST and SLF involvement significantly differed between symptomatic and asymptomatic patients (z = 3.40, P = .0007 and z = 2.85, P = .004, respectively)
**Morrison M.A.**	2016	CAN	To address the influence of technical factors on fMRI and DCS concordance	14	38,6 (-)	14	Higher concordance values and lower between-patient variability for motor mapping (sensitivity: 0.85 ± 0.08; specificity: 0.81 ± 0.07) vs. language mapping. The difference in concordance for motor and language mapping was statistically significant for sensitivity (p < 0.05), but not for specificity
**Tyndall A.J.**	2017	SUI	To analyze the feasibility and limitations of presurgical fMRI for motor and speech maps	491	44,8 (-)	290	BOLD-activation was significantly higher for motor tasks than speech tasks (95,8% vs 81.6%)
**Wongsripuemtet J.**	2018	USA	To compare rs-fMRI with tb-fMRI for localizing the SMA	66	40,8 (18-75)	62	SMA was identified in 75.8% using the left-hand motor ROI, 75.8% using the right-hand motor ROI, 95.5% using the bilateral hand motor ROIs, 27.3% using the left orofacial ROI, 25.8% using the right orofacial ROI, and 34.8% using the bilateral orofacial ROIs. In the tb-MRI group, the SMA was identified in 81.0% scans using the left-hand motor ROI, 90.5% scans using the right-hand motor ROI, 95.2% scans using the bilateral hand motor ROIs, 21.4% scans using the left orofacial ROI, 33% scans using the right orofacial ROI, and 45.2% scans using the bilateral orofacial ROIs
**Liouta E.**	2019	GR	To validate the functional relevance of rs-fMRI by investigating the association between rs-fMRI and preoperative motor and language function performance	69	50 (18-78)	49	Paretic patients showed significantly (p = 0.01) decreased BOLD signal in ipsilesional precentral gyrus when compared to contralesional one. Significantly (p < 0.01) lower BOLD signal was also observed in ipsilesional precentral gyrus of paretic patients when compared with the non-paretics. In asymptomatic patients, a strong positive correlation (r = 0.68, p < 0.01) between ipsilesional motor cortex BOLD signal and contralesional finger tapping performance was observed. Patients with aphasia showed significantly (p = 0.01) decreased rs-fMRI BOLD signal in left BA 44 when compared with non-aphasics. In asymptomatic patients, a strong positive correlation (r = 0.72, p < 0.01) between BA 44 BOLD signal and phonological fluency performance was observed
**Weiss Lucas C.**	2020	DE	To assess the congruency of nTMS and fMRI with DCS	36	56 (-)	26	Significantly smaller Euclidean distances (11.4 ± 8.3 vs. 16.8 ± 7.0 mm) and better spatial overlaps (64 ± 38% vs. 37 ± 37%) between DCS and nTMS compared with DCS and fMRI

BOLD, blood-oxygen-level-dependent; CST, corticospinal tract; LAD, lesion to activation distance; ROI, region of interest; SG, sentence generation; SLF, superior longitudinal fasciculus; SMA, supplementary motor. area, WG, word generation.

**Table 3:** Extraction of the most significant studies investigating MEG’s role as a preoperative mapping tool.

Author	Year	Country	Aim of study	N. of patients	Mean age (y/o)	N. of gliomas	Results
**Rezai A. R. et al.**	1996	USA	To integrate the MEG sensorimotor mapping information into a stereotactic database, using CT, MRI scans, and digital angiography	10	38 (-)	7	Excellent qualitative anatomic agreement between MEG and MRI in every patient. Quantitative analysis demonstrated correlation with an absolute distance error of < 2.5 mm
**Ganslandt O. et al.**	2003	DE	To evaluate the utility of preoperative MEG	119	46,3 (5-77)	111	46.2% of patients (tumors in sensorimotor-, speech- and language-related areas) were not considered for surgery due to the MEG findings; only 6.2% of patients of the surgical group developed neurological consequences
**Oishi M. et al.**	2003	JPN	To assess the clinical value of MEG in localizing the primary hand motor area and evaluating cortical distortion of the sensorimotor area	14	38,6 (22-66)	8	MEP by DCS confirmed the accuracy of MEG in five patients. The medial-lateral distances of equivalent current dipole locations between the primary sensory and motor components were significantly shorter in these patients than in the control patients without intracranial tumors (p = 0.05)
**Schiffbauer H. et al.**	2003	DE, USA	To compare MEG with intraoperative ECM for the localization of sensorimotor cortex	224	42 (13-82)	190	MEG-based mapping was technically successful 97% with hand digits for both tumor-affected and unaffected hemispheres, lip localization was possible in more than 90% of cases. The 3D distance between somatosensory sites on MEG and somatosensory sites on intraoperative DCS was a mean of 20.5 +/- 1.5 mm, therefore still unsatisfying. 81% of the patients left the hospital neurologically unchanged or improved despite the radical tumor resection strategy
**Grummich P. et al.**	2006	DE	To compare the language localizations acquired with MEG and fMRI	172	- (-)	110	77% congruence between MEG and fMRI
**Korvenoja A et al.**	2006	FIN	To prospectively evaluate MEG and fMRI, as compared with intraoperative cortical mapping, for the identification of the central sulcus	15	44,6 (25-58)	13	MEG proved to be superior in localizing the central sulcus compared to fMRI
**Kirsch H. et al.**	2007	USA	To predict the location of mouth motor and sensory cortex	13	42,1 (28-61)	13	Although presenting a considering anterior (17.9 mm) and lateral (16.2 mm) shift for sensory and motor DCS sites in relation to SSEF, respectively, MEG was able to match up direct stimulation
**Tarapore P.E. et al.**	2012	USA	To compare the accuracy of nTMS motor mapping with both DCS and MEG	24	45 (27-70)	23	The median distance between TMS and MEG mapped motor sites was 4.71 ± 1.08 mm. 3 of 24 patients developed new, postoperative paresis of the upper extremity. Two of these patients significantly improved after 3 months
**Niranjan A. et al.**	2013	USA	To assess sensory cortex localization in patients with brain tumors, arteriovenous malformations, and epilepsy and its effect on outcomes after neurosurgical procedures	45	47 (16-77)	13	No association between age and somatosensory peak latency; 100% postoperative retention of somatosensory function
**Zimmermann M. et al.**	2019	DE	To investigate if a combination of MEG and fMRI increases the accuracy in the identification of sensomotory areas	13	49,5 (32-76)	11	Overall concordance was found between MEG and fMRI with a median dispersion of 10mm
**Zimmermann M. et al.**	2019	DE	To investigate if a combination of MEG and fMRI increases the accuracy in the identification of speech areas	18	46,4 (21-74)	15	Overall concordance was found between fMRI and MEG with a median dispersion of 10 mm

DCS, direct cortical stimulation; ECM, electrophysiological cortical mapping.

### 3.1 nTMS

Since its introduction in 1985 by Barker et al., nTMS has been used in specialized neurosurgical centers to improve tumor resections in eloquent brain areas without increasing the risk of postoperative neurological deficits. The safety and tolerability of nTMS for neurosurgical planning was demonstrated by Tarapore et al., who reported no seizures or other adverse events in a multicenter study of 733 brain tumor patients (17).

**Figure 4:**
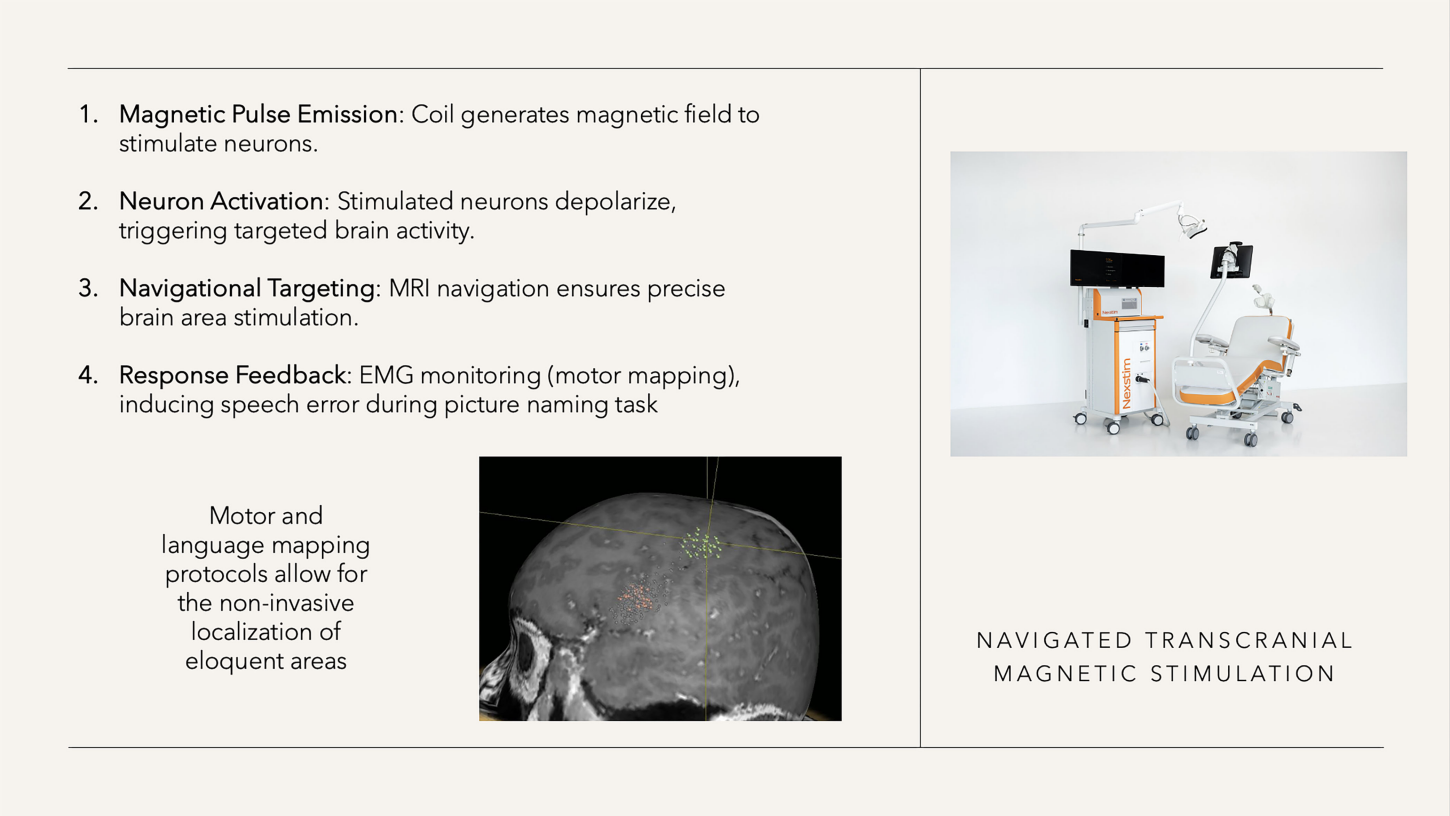
nTMS characteristics (right-sided figure with kind permission from Nexstim).

This non-invasive brain stimulation technique uses a coil to generate a variable magnetic field (strength ranging between 2-3 Tesla), inducing changing electrical currents in the brain through electromagnetic induction (**Figure 4**). (18–20) Due to the brain’s non-uniform structure, current distributions may be distorted, leading to the development of technical methods to reduce spatial errors. Various techniques, including stereotactic positioning with optical navigation systems, have been employed for precise targeting specific brain regions, resulting in navigated (n)TMS (18–22).

The motor resting threshold (RMT) is a well-known standardized parameter in neurophysiology and describes the individual excitability of the motor system (23–25). It varies between individuals (e.g. due to anatomical differences that influence the distance between the coil and cortex). The RMT is often used, for example, to assess the intraindividual excitability between the hemispheres or to adjust the stimulation intensity for motor and language mappings (26, 27).

#### 3.1.1 nTMS motor mapping

In 2009, Picht et al. reported one of the earliest uses of an electromagnetic navigation system to position the nTMS coil in a larger, prospective patient cohort (28). In this and other studies, the accuracy was validated in comparison with direct cortical stimulation (DCS) (mean deviation from DCS 2-4mm) (29–32). A direct comparison between nTMS- and fMRI-determined motor areas with DCS showed better agreement for the nTMS motor mapping (33). When using nTMS, it is important to consider the calculated stimulation accuracy after co-registration with MRI and to accept only a calculated tolerance range of ≤2 mm. Failure to comply has been shown in a study to result in individual cases with significant deviations compared to DCS (34).

Many institutions have explored the clinical benefits of nTMS in surgical planning due to its spatial accuracy in creating three-dimensional functional maps (8, 35, 36). In a study by Rizzo et al., neurosurgeons reported that nTMS motor mapping provided valuable anatomo-functional information in 71% of cases and influenced operative techniques in 29% of surgeries, leading to changes in surgical strategy (37). Frey et al. also observed changes in surgical approaches for over half of patients mapped with nTMS, resulting in a significant increase in gross total resections from 42% to 59% (38). A comprehensive/summarizing meta-analysis by Raffa et al. showed a reduction in postoperative motor deficits (OR = 0.54), an improvement in the extent of resection (GTR OR: OR = 2.32) and an optimization of craniotomy size (-6cm^2^) and operation duration (-10min) in motor eloquent brain tumors (39).

#### 3.1.2 nTMS-based diffusion tensor imaging fiber tracking of the CST

The idea behind combining nTMS motor maps and DTI tractography was to provide a function-based, individual tractography of the essential tracts of the CST. Conventional tractography algorithms often failed to visualize the CST due to tumor mass effects and peritumoral edema, which has now become possible through the integration of functional nTMS data (40, 41). In detail, Frey et al. defined the term “FA threshold” which enables an individually tailored representation of the CST (40).

In 2014, Conti et al. introduced a technique for somatotopic DTI tractography based on the somatotopic nTMS motor mapping of different muscles (42). The preoperative tractography analyses of 20 patients were integrated into the intraoperative neuronavigation and the accuracy was confirmed using subcortical stimulation. Thus, nTMS-based tractography provides a reliable anatomic and functional characterization of the motor pathway. Rosenstock et al. proposed a manual for standardized nTMS-based tractography confirming the reliability and user-independence of nTMS-based tractography. Even inexperienced users are able to perform the tractography and determine the tumor-tract distance reliably (ICC > 0.9) (43). The superiority in terms of accuracy of nTMS-based tractography compared with conventional tractography was confirmed with direct stimulation (44). In comparison to fMRI as another method for function-based tractography, nTMS-based tractography showed higher plausibility rate, whereas fMRI-based tractography falsely visualized posterior pathways that are presumably functionally more associated with the sensory system (45).

#### 3.1.3 Prognostic value of nTMS mapping/nTMS-based tractography for the motor function

Takakura et al. found a correlation between hotspot-tumor distances and postoperative upper-extremity motor function recovery (46). Patients with greater distances (>10mm) showed better grip strength recovery at 3 months. Krieg et al. demonstrated the prognostic value of nTMS functional mapping in influencing surgical strategy and reducing iatrogenic damage (47, 48). They compared a cohort of 100 nTMS-guided tumor resections with a historical control group and found lower residual tumor rates, improved motor function, smaller craniotomies, and increased eligibility for chemotherapy and radiation therapy in the nTMS group.

Moser et al. and Hendrix et al. further validated the prognostic significance of nTMS positive motor areas anterior to the motor cortex but achieved conflicting results (12, 49). However, when simulating premotor areas care must be taken to ensure that the motor cortex is not stimulated incidentally, which can lead to false-positive results (50).

Several authors investigated the white matter integrity of the CST by analyzing the tumor-tract distance (TTD) as well as the fractional anisotropy (FA) and the apparent diffusion coefficient (ADC). Interestingly, Rosenstock et al. demonstrated that a lower average FA within the affected CST as well as a higher average ADC were associated with deteriorated postoperative motor function (43). In a prospective cohort of 113 patients, no new postoperative motor deficit occurred when the TTD was greater than 8 mm and the precentral gyrus was not infiltrated (27). The relevance of the TTD was confirmed by Sollmann et al. who found that no patient with a TTD ≥ 12 mm suffered from new surgery-induced permanent paresis (51).

Finally, Rosenstock et al. proposed a bicentric-validated risk stratification model using nTMS motor maps and nTMS-based tractography to predict postoperative motor outcomes (short-term and long-term after 3 months) in glioma patients (27, 52). In a study of 278 patients, they found no new permanent deficits when the tumor was > 8 mm distant from the CST and did not infiltrate the precentral gyrus. By assessing the integrity of the CST (by measuring the fractional anisotropy) and of the motor system (by determining the RMT), they could calculate the patients’ individual risk of a new postoperative motor deficit. Further studies demonstrated the clinical and prognostic value of nTMS-based DTI for motor eloquent tumors (53–56). Ivren et al. demonstrated that nTMS-based prediction outperforms an anatomy-based risk assessment (only using structural MRI data) and better predicts the extent of resection (57).

Despite the many published (prospective) studies, no RCT data is yet available since the analysis of the first RCT on the use of preoperative nTMS mapping is pending (NCT02879682).

#### 3.1.4 nTMS language mapping

One of the first experiences of repetitive navigated transcranial magnetic stimulation (rnTMS) was reported by George et al., Herwig et al., and Lioumis et al. for language mapping by inducing temporary disruptions in specific brain regions to identify language-related areas (19, 58, 59). Several stimulation algorithms have been studied over the years, resulting in a recommendation on rnTMS language mapping stated by an international consortium of experts (26): stimulation frequency 5-7 Hz, applied pulses 5-7, picture presentation time 500-700ms, time between picture presentation and stimulation 0-300ms. However, rnTMS language mapping with stimulation frequencies up to 50 Hz was also evaluated in healthy subjects to improve the reliability (60).

In 2013, Picht et al. conducted the first study comparing the use of rnTMS for functional mapping of language areas in combination with direct electrical stimulation (DES) during awake surgery to analyze its reliability in identifying language regions in patients with left-sided lesions (61). The overall evaluation of the following studies showed a high (and therefore clinically significant) sensitivity (range: 63%-97%) and negative predictive value (range: 74%-99%) (62–64). The accuracy appears to be higher for the frontal language regions (Broca’s area), where a sensitivity and NPV of 100% were achieved in two independent studies (47, 61). Thus, tumor resections in rnTMS language-negative areas can be performed with a very low risk for the occurrence of new postoperative language deficits. Some centers even decide not to perform awake craniotomies in these cases, however, the proximity to language-associated tracts must be taken into account (65).

Disadvantages of rnTMS language mapping are the average and highly variable specificity (13%-98%) and positive predictive value (24%-69%). Schwarzer et al. showed a very high rate of false-positive mapping results in patients with neurocognitive deficits and preoperative language impairment, so that rnTMS language mapping should not be performed in cases with baseline error rates >28% (66).

Similar to the nTMS-based DTI FT of the CST, the combination of rnTMS language mapping with DTI studies has been utilized to visualize the language network by emphasizing the rnTMS-associated functional cortico-subcortical tracts. Several studies have explored the integration of rnTMS language mapping and rnTMS-based DTI FT for surgical planning, showing consistent results (67–70). In conclusion, rnTMS-based DTI FT has proven to be valuable in guiding surgical decisions and reducing residual tumor volume (71). However, it should be noted that tractography solely based on rnTMS seeds is not recommended since this will lead to unplausible tractography maps (72). According to the current state of clinically available tractography algorithms, it is rather recommended to first identify pathways based on anatomical ROIs and then integrate the rnTMS information to investigate functional corticosubcortical pathways (67, 73).

### 3.2 fMRI

**Figure 5:**
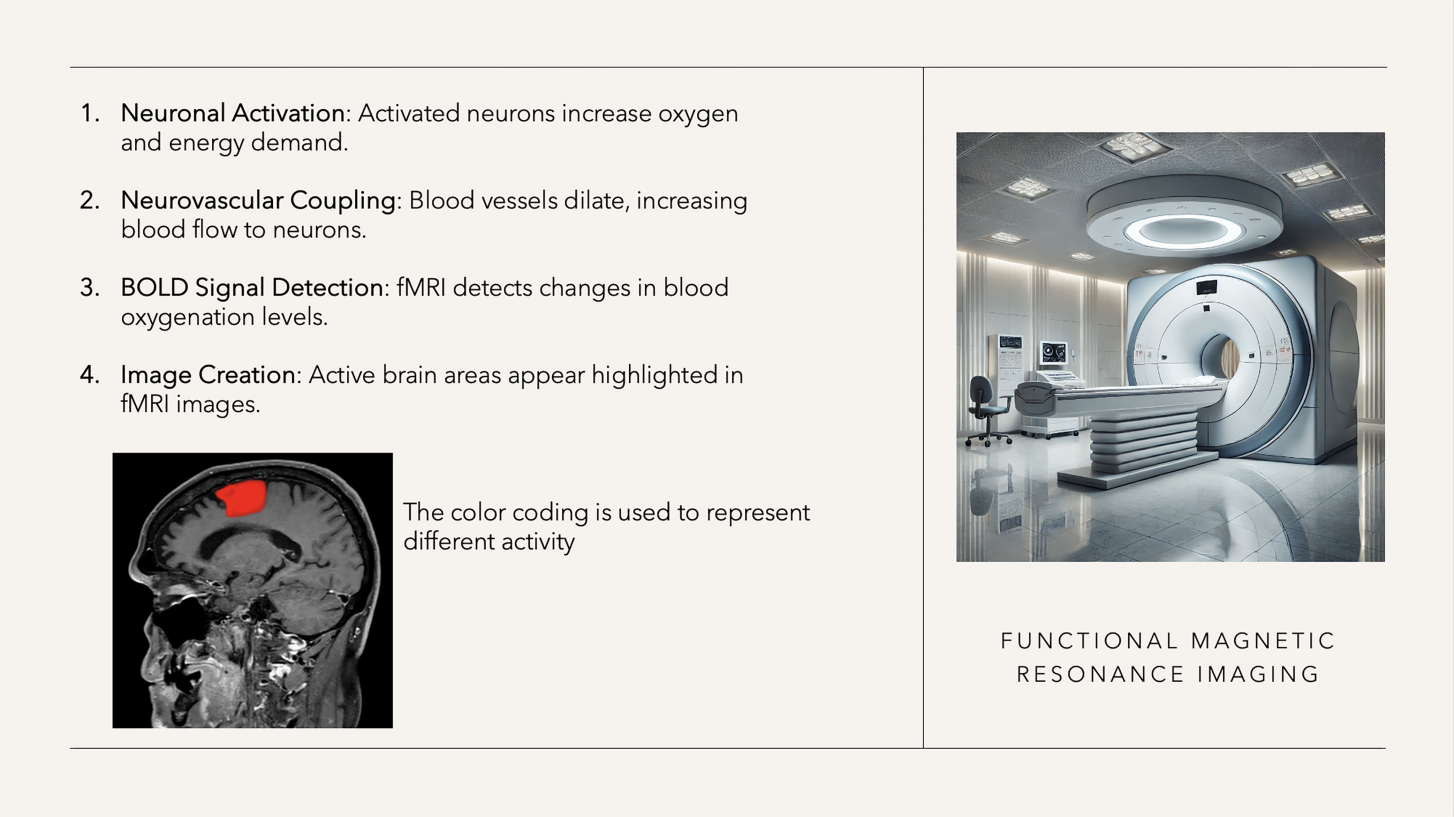
fMRI characteristics.

fMRI is a crucial preoperative mapping tool that identifies cortical and subcortical activity by detecting changes in brain vascular flow through the BOLD (blood-oxygen-level-dependent) effect, which associates cerebral blood flow with neural activity via neurovascular coupling (**Figure 5**) (74). Since the introduction of the BOLD protocol in 1990 by Ogawa and colleagues (75), fMRI has become a valuable tool in neurosurgery and functional neuroscience due to its ability to accurately show brain regions involved in processing internal or external stimuli (76). This oxygenation-sensitive imaging technique provides precise spatio-temporal accuracy by capturing changes in oxyhemoglobin and deoxyhemoglobin levels linked to neuronal activity (77). While other protocols have been suggested, discussing their advantages and disadvantages is beyond the scope of this review (78).

#### 3.2.1 Task-based fMRI motor mapping

In 1998, Pujol et al. reported the first cases of using three-dimensional fMRI mapping to identify the central sulcus as part of preoperative planning for patients with space-occupying lesions (79). The most commonly used task-based motor mapping fMRI methodology is based on conscious, repetitive motor activations in response to the examiner’s instructions (7, 80–82). Such protocols are based on BOLD signal fluctuations between task-induced and control states, whose correlation with intraoperative mapping data has been shown in several studies (83).

Several studies confirmed the clinical utility of tb-fMRI motor mappings, e.g. in selecting the appropriate surgical approach, minimizing craniotomy size (and thus brain exposure), and reducing postoperative neurological complications caused by tumor-induced alterations in expected anatomy and function (84). The studies on neuroplasticity showed varying- and partially contradictory - results. For instance, Baciu et al. (85) and Alkadhi et al. (86) used a block paradigm to study neural plasticity in patients with primary brain lesions, focusing on hand, leg, and mouth movements. They observed that the most common tumor-induced neural plasticity involves inter-hemispheric reorganization, with only few cases of intra-hemispheric reorganization or activation outside the primary motor area. In contrast, Nelson et al. (87) demonstrated varying levels of neural reorganization in the supplementary motor area (SMA), including ipsilateral SMA activation, bilateral activation, and different grades of reorganization. In the small cohort of 12 patients, they found a significant association between the distance between the tumor and SMA region (5mm) and the occurrence of new postoperative neurological deficits (paralysis and speech impairment/mutism).

Although many studies on the clinical applicability of task-based fMRI motor mappings exist, this technology has two significant drawbacks. Firstly, there is no established risk stratification that can differentiate between eloquent-activated areas (where resection leads to neurological deficits) and co-activated areas (where resection results in no or transient deficits). Secondly, several studies have shown that fMRI signal analysis near brain tumors is distorted due to neurovascular uncoupling (i.e. associated with vasogenic edema) and has led to inaccuracies compared to the gold standard (DCS) (88–93). Thus, the motor cortex could not be identified in 18% of patients and the distance between the DCS hotspot and fMRI hotspot was frequently >1cm, which hindered adequate surgical planning (94–96). Ultimately, the sensitivity and specificity of fMRI were 61.7% and 93.7% (94, 97, 98). In DCS-based comparative studies, fMRI was significantly inferior compared to nTMS in terms of identifying motor eloquent areas (33, 95, 99). In an effort to improve subcortical spatial orientation during surgical resection, fMRI-identified areas were used as ROIs for DTI tractography of the corticospinal tract (100, 101). In a comparative study, fMRI-based tractography was significantly inferior to nTMS-based tractography in terms of plausibility and occurrence of aberrant fiber pathways. Particularly when the tumor was in close proximity to functional areas, changes in BOLD signal physiology occurred, highlighting nTMS-based tractography as the method of choice (45).

#### 3.2.2 Resting-state-fMRI motor mapping

Dierker and colleagues were among the first to compare rs-fMRI with tb-fMRI acquisition (102). By detecting task-negative states, rs-fMRI enables researchers to identify spontaneous fluctuations in BOLD signals, forming resting-state networks (RSN) (103). The main benefit of rs-fMRI is the ability to generate functional maps even in uncooperative individuals (such as children or incompliant adults), as long as they remain calm during the scan (104–108). rs-fMRI tends to additionally detect network-based associated regions (such as the sensory cortex) (109), resulting in the detection of larger areas but also raising further questions about their surgical relevance and usability.

The aforementioned disadvantages of tb-fMRI are even more pronounced here, as rs-fMRI mapping relies essentially on identifying preserved anatomical landmarks that may be altered by the lesion. Furthermore, the acquisition times for rs-fMRI can still be too long at times, although interesting new advances through machine learning (ML) and artificial intelligence show promising results (110).

#### 3.2.3 fMRI language mapping

Despite the complexity of the language network, the initial experiences date back to 1999 by Ruge et al., where a good concordance between tb-fMRI activated areas and DCS was found in 5 patients (111). Current standard paradigms include picture-naming tasks and word-listening tasks, which are also utilized in assessing neuroplasticity (112, 113). Although many studies have reported positive experiences with fMRI for investigating the language network (13, 114, 115), very similar limitations exist as with fMRI motor mapping. Particularly when the tumor is located very close to eloquent areas (<1cm), involves high-grade gliomas with blood-brain barrier disruption and perifocal edema, or well-vascularized tumors, authors have found significant inaccuracies (sensitivity: 37.1%, specificity: 83.4%) (116, 117). In another study involving 50 patients with left-hemispheric gliomas, in some cases, a right-hemispheric language dominance was mistakenly indicated, which was explained by a reduced perilesional fMRI signal due to lesion-induced neurovascular uncoupling (118).

Studies on rsfMRI have shown that even uncooperative patients could be examined, leading to the detection of co-activated areas and thus overcoming this intrinsic limitation of tb-fMRI. However, this also complicates the interpretation of results, as it remains difficult to differentiate between eloquent and non-eloquent, co-activated areas (119–121). rsfMRI analyses should be interpreted with caution, especially when tumors alter the anatomical landmarks as well as the functionality of the RSN, leading to incorrect results (122).

### 3.3 MEG

**Figure 6:**
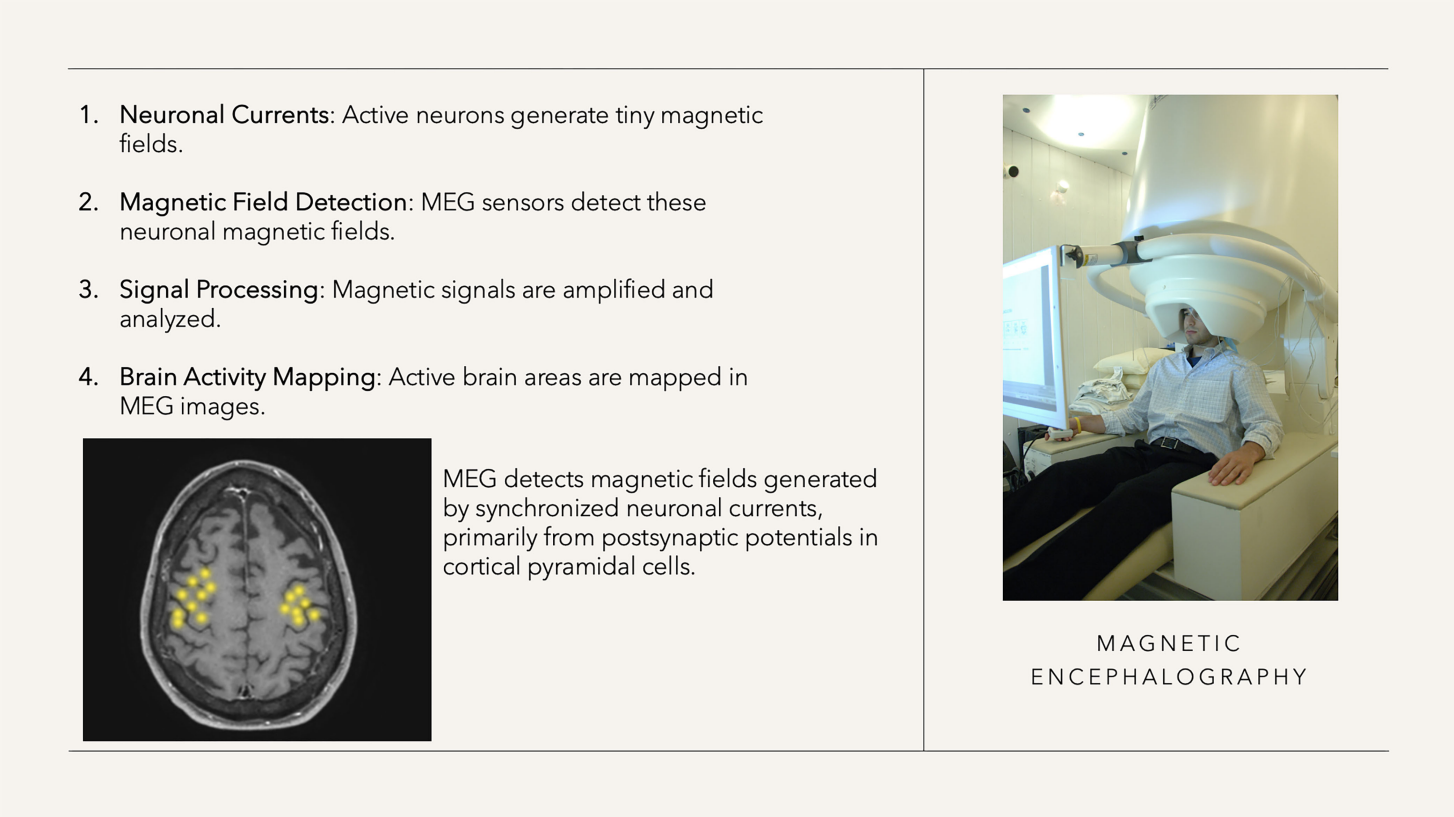
MEG characteristics (right-sided figure approved by the National Institute of Mental Health, Department of Health and Human Services).

Since its inception in 1968 by Illinois physicist David Cohen, MEG is a relatively new technology used in cognitive brain sciences, oncological neurosurgery, and neurotherapy with real-time neurofeedback (123, 124). It operates on high-sensitivity arrays of SQUIDs (superconducting quantum interference devices) magnetometers to capture and map synchronized electrical brain activities by recording magnetic fields from activated synapses (**Figure 6**). Despite limited use in neurosurgery due to cost and infrastructure requirements, MEG-based brain mapping offers advantages over other techniques, such as less distortion compared to EEG and superior temporal resolution compared to fMRI (125). While MEG mapping is not widely studied as a standalone technique in medical literature, it is often combined or compared with other methods like nTMS and fMRI for preoperative planning in neuro-oncology.

#### 3.3.1 MEG motor mapping

The first clinical experience with magnetoencephalography (MEG) dates back to 1995, when Gallen et al. attempted to utilize somatosensory evoked potentials to predict the pattern of phase reversals observable intraoperatively on the cortical surface in six patients with various pathologies. They identified a correlation across the entire cohort, with an average deviation of 8 mm compared to the intraoperative findings (126). In 1996, Rezai et al. reported on the integration of MEG-derived brain mapping with various imaging techniques to develop a preoperative algorithm for navigating during the resection of eloquent lesions in the sensorimotor cortex (127). Schiffbauer et al. used MEG to investigate the spatial relationship between functional eloquent areas and tumors, finding functional activity in 18% of grade 2 tumors, 17% of grade 3 tumors, and 8% of grade 4 tumors (128).

In numerous studies, MEG results have been compared with fMRI analyses and validated in a few studies using intraoperative DES. Overall, there was good agreement (and partial superiority) with fMRI analyses (129–131), but a mean deviation of DES-identified motor hotspots of 12.5mm, which poses a significant limitation for neurosurgical operation planning (132–135). This spatial deviation poses a particular challenge for precise neurosurgical planning. MEG localization often relies on the mapping of somatosensory evoked fields (SEFs) rather than signals from the motor cortex. SEFs localize the somatosensory cortex, with sources located deeper in the central sulcus, while direct electrical stimulation (DES) targets the crown of the precentral gyrus to identify motor hotspots. This anatomical variance between somatosensory and motor cortex activation contributes to the observed spatial discrepancies and must be taken into account (136, 137). The reliability of motor mapping for planning brain tumor resections is not compromised in centers with experience in MEG analysis, as the deviation between the activity shown by MEG and the DES stimulation was generally ≤10 mm (133).

Tarapore et al. compared nTMS with MEG and DES for cortical motor mapping in patients with malignant lesions near the central region. While a direct comparison between MEG and DES was not conducted, the mean distance between nTMS and MEG imaging motor sites was 5 mm, and between nTMS and DES motor sites was 2 mm, suggesting a strong alignment between MEG and nTMS (34). In one case of the study, no MEPs could be elicited by preoperative nTMS or intraoperative DCS; however, there was motor-functional MEG activity within the lesion. Postoperatively, a permanent motor deficit was evident in this case. This individual case suggests that MEG may be valuable in situations where neither nTMS nor DCS is able to induce MEPs.

In a study of 119 patients with gliomas, the relative spatial relationship between the tumor and eloquent brain areas (specifically sensory-motor, visual, and language areas) was analyzed (138). In high-risk cases with distances <5mm, surgery was avoided, which occurred in 46% of patients. Although the rate of postoperative neurological deficits was low at 6%, it raises questions about whether some non-operated patients may have been suitable for resection, potentially missing the opportunity for longer overall survival.

In a feasibility study, the language associated areas detected by MEG were used as ROI and combined with diffusion tensor imaging (DTI) to visualize the arcuate fasciculus in patients with lesions in language regions. However, the clinical relevance and benefits for surgical planning and the correlation with postoperative outcomes have yet to be investigated (139).

#### 3.3.2 MEG language mapping

In contrast to MEG motor mapping, there are very few studies available on MEG-based language mapping, which primarily focus on interhemispheric language lateralization. In one of the initial studies, Grummich et al. combined fMRI and MEG to investigate language localization in 172 patients with brain lesions (140). They found a high level of agreement between the two techniques in identifying language sites (77% congruence), with only a small percentage showing differences (4%). MEG mapping was shown to be superior in nearly half of the cases (in which the BOLD signal was suppressed, particularly in glioma patients). Another study investigating language localization was conducted by Szymanski et al. in 2001, who demonstrated a general left asymmetry in right-handed neurosurgical patients through MEG mapping, as well as a right asymmetry in two patients with confirmed right hemispheric language dominance via the amobarbital test (141).

While the Wada test is nowadays used by only 12% of epilepsy centers for language lateralization assessment, it has been considered the “gold standard” for preoperative determination of language dominance (142). Doss et al. validated MEG-based language localization with the Wada test, reporting a relatively low concordance rate (69%) between the two methods (143). Ota et al. (144) compared MEG and fMRI with NIRS (near-infrared spectroscopy) and the Wada test for language lateralization, finding that fMRI and NIRS had higher sensitivity and specificity in patients with typical hemispheric dominance, while NIRS showed better specificity in patients with right language lateralization (144). However, the authors emphasized the individual, high technical requirements (device differences, patient characteristics such as brain edema and movement artifacts) associated with MEG, fMRI and NIRS, which make (routine) use difficult (145). In a comparative study between MEG and nTMS language mapping for determining language dominance, a 64% agreement was found. However, no validation with the WADA test was performed which limits the significance of the study (146).

## 4 Discussion

The management of CNS tumors involves intricate preoperative planning that considers individual patient characteristics, especially their functional neuroanatomy, to achieve maximal resection while preventing new neurological deficits. Most studies on preoperative mapping were fMRI-based (n=56), followed by nTMS (n=48), with significantly fewer studies on MEG (n=26). Motor mapping is well established in routine practice, with nTMS-based motor mapping proving to be the most accurate. Studies on preoperative language mapping showed highly heterogeneous results with varying levels of agreement with direct electrical stimulation (DES) during awake craniotomies (**Table 4**).

**Table 4:** Benefits, drawbacks and potential future studies of nTMS, fMRI, and MEG in preoperative mapping.

Technique	Benefits	Drawbacks	Future Study Suggestions
**nTMS**	• Highest accuracy for motor mapping compared to DCS • Enables risk stratification/predicts motor outcome • Combination with DTI allows individual tractography • Reduces postoperative motor deficits and enhances EOR • High NPV for language mapping	• Requires trained operators • Prone to false positives in language mapping (especially in patients with neurocognitive deficits)	• Multicenter RCTs may validate prognostic impact and standardize protocols across centers • Focus on language mapping in specific patient populations, particularly those with preoperative neurocognitive impairments, may determine reliability thresholds • Development of tailored mapping tasks and novel stimulation patterns • Evaluation of tractography-guided stimulation • Investigating further applications of TMS-EEG
**fMRI**	• Non-invasive with good spatial resolution for motor and language mapping • tb-fMRI aids in planning surgical approach, minimizing craniotomy size • rs-fMRI useful for patients unable to perform tasks	• Prone to false positives and negatives due to neurovascular uncoupling near tumors • Less accurate than nTMS for motor mapping • Task compliance required for tb-fMRI • Limited accuracy for subcortical mapping • Limited applicability of language fMRI for resection planning	• Investigating machine learning and AI algorithms to better differentiate between eloquent and non-eloquent, co-activated areas in fMRI data • Comparing rs-fMRI results with intraoperative DCS to validate resting-state network identification in uncooperative patients (e.g., pediatric or aphasic) • Evaluate rs-fMRI reliability in cases of neurovascular uncoupling to develop improved analytical techniques for peritumoral mapping
**MEG**	• High temporal resolution and minimal distortion • Effective for motor and language mapping, even detecting critical sites overlooked by other methods • Useful for complex cases as a complementary modality with nTMS	• High cost and infrastructure requirements • Limited availability and technically demanding • Spatial deviation from DCS for motor mapping (~12.5mm), impacting precision • Less evidence on standalone use and validation compared to nTMS and fMRI	• Examining cost-effective MEG models and data-processing methods to make MEG more accessible • Investigating MEG’s unique ability to detect critical motor sites missed by other modalities, especially in complex cases where conventional mapping is inconclusive • Focusing on MEG-based language mapping should establish a standardized approach for preoperative planning

DCS, direct cortical stimulation, DTI, diffusion tensor imaging, EOR, extent of resection, NPV, negative predictive value; rs-fMRI, resting-state fMRI, tb-fMRTI, task-based fMRI.

### 4.1 Preoperative motor mapping

In previous studies, nTMS-based motor mapping was found to be the most accurate method compared to DCS for both upper and lower extremities. In contrast, fMRI-based mapping was not as precise and exhibited a greater deviation from the DCS-based motor hotspot (95, 99, 147, 148). The nTMS motor mapping is based on single-pulse stimulation, and only if the stimulation induces a motor-evoked potential is it considered positive. In contrast, with fMRI and MEG, areas are also considered activated that would be assigned to the secondary motor system (such as movement planning in prefrontal areas). However, resections in the co-activated areas do not necessarily lead to motor deficits, which must be taken into account when interpreting fMRI- and MEG-based motor maps to avoid unnecessarily restrictive resections and compromising oncological outcomes.

Besides the high standardization and interrater reliability, an nTMS risk stratification has been developed based on motor mapping and tractography, which further strengthened its application in routine practice (27, 52). In detail, the risk for a new or aggravated motor deficit can be predicted preoperatively, allowing for individual patient counseling and surgical planning. No new motor deficits occurred in patients with a TTD > 8mm and without motor cortex infiltration, so in these cases, gross total resection (GTR) is recommended, and the use of IOM is not necessarily required. Although intraoperative validation studies have shown high agreement with subcortical stimulation, the use of IOM is strongly recommended for resecting motor eloquent gliomas with a tumor tract distance (TTD) ≤ 8 mm to preserve the integrity of the motor system, as it is not possible to account for all intraoperative influences like brain shift (149).

In addition to improving clinical outcomes and preoperative planning, the cost-effectiveness of nTMS motor mapping has been demonstrated (145). Corresponding calculations for fMRI- and MEG-based motor mappings are not available.

The acquisition costs for an MEG system are substantial and comparable to those of a 3T MRI scanner; however, recent technological advancements, such as helium recycling, have contributed to reducing long-term operating costs. Nevertheless, the acquisition and operating costs still exceed those of an nTMS system (150). Furthermore, the process of analyzing dipole sources in MEG is automated. The operation (and associated costs) of an MEG device after its installation can be streamlined just like that of fMRI, which could enhance its feasibility and accessibility in hospitals with adequate infrastructure (151).

In addition to SEFs, alternative MEG-based methods such as corticomotor coherence (CMC) and beta band suppression (event-related desynchronization or ERD) have been investigated for mapping motor cortex function. While these techniques provide valuable insights into the dynamics of the motor network, they often result in complex data analysis and frequently yield spatially dispersed activation patterns. In contrast, it has been shown that the nTMS motor mapping provides high spatial resolution with excellent intra- and interindividual reliability *(* 152 *).*

Finally, ML algorithms were recently applied to detect previously unrecognized patterns. For example, Shams et al. recently developed an ML algorithm to predict motor deficits in glioma patients based on quantitative parameters such as FA, ADC, axial, and radial diffusivity examined along the tract statistics (153). However, these AI-supported methods are highly technical and require considerable technical expertise with promising - but still insufficient - accuracy, which has so far significantly limited their routine use and dissemination in patient care.

### 4.2 Preoperative language mapping

Preoperative language mapping is known to be more challenging to investigate, because the language network is significantly more complex than the motor system and because there is a greater variety of possible language deficits (154). Studies comparing the concordance of the mapping methods with DCS did not show consistent results, thus a standardized preoperative language mapping method or protocol has not been established across centers. In addition to a few studies comparing different mapping methods, there are no multicenter, larger studies demonstrating the superiority or inferiority of the respective techniques. The treatment of patients with language-eloquent brain tumors is characterized by center-specific experience and expertise in the respective mapping method available. The decision-making process between language eloquent brain areas and tumor tissue to be resected is therefore far from standardized.

In studies on rTMS language mapping, a high specificity with a very high negative predictive value was predominantly observed (61, 63, 64, 66, 155–157). Centers with extensive experience in rTMS language mapping decide on the basis of the rTMS mapping results (so-called negative mapping) whether patients should undergo awake surgery. rTMS-negative areas can therefore be safely resected, but care must be taken at the resection margins to avoid damaging the subcortical language network. In a direct comparison between rTMS and fMRI, rTMS mapping showed a higher negative predictive value (rTMS 100% vs. 73% fMRI), indicating a better suitability for preoperative mapping (158–162). In cases of severe neurocognitive impairments or advanced aphasia, rTMS language mapping should not be performed due to increased false-positive results (66). Interpretation of fMRI language mapping is particularly difficult due to frequent coactivation of areas leading to false-positive activations and peritumoral false-negative signal suppression (due to neurovascular uncoupling caused by the lesion).

The mapping technologies can also be used to determine hemispheric language dominance (115, 118, 142, 146). However, as the majority of patients have a left hemispheric dominance, the results of the examination rarely have an impact on the treatment strategy.

## 5 Conclusion

nTMS motor mapping has proven to be superior to fMRI in terms of accuracy (compared to DCS) and clinical applicability. nTMS with nTMS-based tractography allows for standardized preoperative risk stratification to predict short-term and long-term motor outcomes. MEG demonstrates high accuracy in motor mapping; however, its use is limited due to the high costs and technical demands, similar to fMRI. Mapping language function remains challenging with a wide range of agreement with DCS for all mapping methods, thus no standard has been established in clinical routine. rTMS stands out for its high negative predictive value across studies, allowing it to evaluate patients for an awake craniotomy. When interpreting fMRI results, attention must be paid to co-activated but non-eloquent areas (false positives) as well as false negatives due to neurovascular uncoupling, significantly limiting its utility in preoperative planning for both motor and language functions.
