# Supplementary material for: Preoperative mapping techniques for brain tumor surgery: a systematic review
Source: Front Oncol. 2025 Jan 7;14:1481430. doi: 10.3389/fonc.2024.1481430 (PMC11747149; doi:10.3389/fonc.2024.1481430)
Supplement: Supplementary file 1 [file Table1.docx]

| Author | Year | Country | N. of patients | Sex distribution (% M) | Age range (y/o) | Mean age (y/o) | N. of gliomas | JBI |
| --- | --- | --- | --- | --- | --- | --- | --- | --- |
| Rezai A. R. et al. | 1996 | USA | 10 | 60 | - | 38 | 7 | 4 |
| Nakasato N. et al. | 1997 | JPN | 14 | 50 | 25-69 | 49,9 | 10 | 5 |
| Ganslandt O. et al. | 1999 | DE | 50 | 44 | 6-79 | 48,3 | 28 | 7 |
| Firsching R. et al. | 2002 | DE | 30 | 30 | 33-79 | 57,4 | 9 | 6 |
| Jannin P. et al. | 2002 | FRA | 11 | 81,8 | 21-70 | 42,9 | 8 | 4 |
| Ganslandt O. et al. | 2003 | DE | 119 | 55 | 5-77 | 46,3 | 111 | 5 |
| Oishi M. et al. | 2003 | JPN | 14 | 42,9 | 22-66 | 38,6 | 8 | 4 |
| Schiffbauer H. et al. | 2003 | DE, USA | 224 | 57,1 | 13-82 | 42 | 190 | 5 |
| Grummich P. et al. | 2006 | DE | 172 | - | - | - | 110 | 4 |
| Korvenoja A et al. | 2006 | FIN | 15 | 40 | 25-58 | 44,6 | 13 | 7 |
| Kirsch H. et al. | 2007 | USA | 13 | 53,8 | 28-61 | 42,1 | 13 | 5 |
| Nagarajan S. et al. | 2008 | USA | 66 | 54,5 | 12-83 | 39,8 | 53 | 3 |
| Doss R.C. et al. | 2009 | USA | 35 | 49 | 15-60 | 28,9 | 12 | 5 |
| Ota T. et al. | 2010 | JPN | 28 | 42,9 | 14-74 | 37,3 | 10 | 6 |
| Tarapore P.E. et al. | 2012 | USA | 24 | 41,7 | 27-70 | 45 | 23 | 7 |
| Niranjan A. et al. | 2013 | USA | 45 | 48,9 | 16-77 | 47 | 13 | 4 |
| Willemse R. et al. | 2015 | NED | 325 | 51,7 | 4-66 | 29,3 | 40 | 5 |
| Zimmermann M. et al. | 2019 | DE | 13 | 69,2 | 32-76 | 49,5 | 11 | 7 |
| Zimmermann M. et al. | 2019 | DE | 18 | 55,6 | 21-74 | 46,4 | 15 | 6 |
| Piai V. et al. | 2020 | NED | 14 | 42,9 | 22-77 | 42,4 | 13 | 6 |
| Rezaie R. et al. | 2020 | USA | 67 | 42 | 5-37 | 16,1 | 8 | 5 |

**Supplementary materials - Table 1.** Extended table showing the list of all the included studies (MEG) and their numeric features.

| Author | Year | Country | N. of patients | Sex distribution (% M) | Age range | Mean age | N. of gliomas | JBI |
| --- | --- | --- | --- | --- | --- | --- | --- | --- |
| Pujol J. et al. | 1998 | ESP | 50 | 44,3 | 17-76 | 62 | 34 | 7 |
| Schulder M. et al. | 1998 | USA | 12 | - | - | - | 6 | 8 |
| Ruge M.I. et al. | 1999 | USA | 21 | 57,1 | 11-71 | 44 | - | 6 |
| Tomczak R.J. et al. | 2000 | DE | 41 | 70,7 | 22-66 | 41,9 | 34 | 7 |
| Gumprecht H. et al. | 2002 | DE | 15 | - | - | - | 11 | 6 |
| Nelson N. et al. | 2002 | USA | 12 | - | - | - | 11 | 9 |
| Baciu M. et al. | 2003 | FRA | 17 | 52,9 | 30-60 | 41,2 | 12 | 6 |
| Roux F.E. et al. | 2003 | FRA | 14 | 64,3 | 14-68 | 43 | 11 | 8 |
| Majos A. et al. | 2004 | PLN | 33 | 36,4 | 24-71 | 54,7 | 20 | 8 |
| Ulmer J.L. et al. | 2004 | USA | 50 | - | 19-64 | - | 31 | 8 |
| Möller M. et al. | 2005 | DE | 10 | 70 | 27-79 | 42,2 | 8 | 7 |
| Roessler K. et al. | 2005 | AUT | 22 | - | 10-65 | 39 | 22 | 8 |
| Stippich C. et al. | 2007 | DE | 81 | 55,6 | 7-75 | 42 | 58 | 7 |
| Wunderlich A.P. et al. | 2007 | DE | 14 | 50 | 26-51 | 38,7 | 14 | 5 |
| Picht T. et al. | 2008 | DE | 30 | 56,6 | 33-80 | 56,7 | 24 | 8 |
| Xie J. et al. | 2008 | CHN | 26 | 73,1 | 22-56 | 40,8 | 26 | 6 |
| Peck K.K. et al. | 2009 | USA | 26 | 52,1 | 35-71 | 65,4 | 26 | 8 |
| Spena G. et al. | 2010 | ITA | 27 | 63 | 12-62 | 45 | 21 | 8 |
| Forster M.T. et al. | 2011 | DE | 10 | 80 | 20-63 | 41,9 | 10 | 8 |
| Lu J.F. et al. | 2012 | CHN | 11 | 63,7 | 26-58 | 40,2 | 11 | 6 |
| Mangraviti A. et al. | 2012 | ITA | 8 | 50 | 20-72 | 42,6 | 6 | 8 |
| Coburger J. et al. | 2013 | DE | 30 | - | 2-76 | 47,8 | 15 | 8 |
| Kundu B. et al. | 2013 | USA | 49 | 71 | 20-72 | 43 | 43 | 5 |
| Kumar A. et al. | 2014 | IND | 15 | - | 18-66 | 35 | 10 | 6 |
| Mahwash M. et al. | 2014 | DE | 37 | 51 | 16-78 | 48 | 23 | 7 |
| Trinh V.T. et al. | 2014 | USA | 214 | 51 | 18-74 | 44 | 214 | 7 |
| Zacà et al. | 2014 | USA | 12 | 41,6 | 25-67 | 43,5 | 12 | 6 |
| Bailey P.D. et al. | 2015 | USA | 76 | 53,9 | 15-78 | 47,4 | 70 | 7 |
| Ille S. et al. | 2015 | DE | 27 | 66,7 | 24-74 | 46 | 24 | 8 |
| Kuchcinski G. et al. | 2015 | FRA | 40 | 55 | - | 39,5 | 40 | 8 |
| Cochereau J. et al. | 2016 | FRA | 98 | 56,1 | - | 40,5 | 98 | 7 |
| Morrison M.A. et al. | 2016 | CAN | 14 | - | - | 38,6 | 14 | 8 |
| Roder C. et al. | 2016 | DE | 12 | 50 | 18-76 | 39 | 11 | 7 |
| Sair H.I. et al. | 2016 | USA | 49 | 63,3 | 18-69 | 39,8 | 48 | 7 |
| Schneider F.C. et al. | 2016 | FRA | 19 | 73,7 | 12-77 | 46 | 9 | 8 |
| Dierker D. et al. | 2017 | USA | 38 | - | - | - | - | 7 |
| Fang S. et al. | 2017 | CHN | 35 | 74,3 | - | 40 | 35 | 7 |
| Gębska-Kośla K. et al. | 2017 | PLN | 10 | 50 | 22-55 | 36,3 | 10 | 6 |
| Qiu T.M. et al. | 2017 | CHN | 30 | 63,3 | 19-70 | 42,5 | 28 | 7 |
| Tyndall A.J. et al. | 2017 | SUI | 491 | 54,2 | - | 44,8 | 290 | 7 |
| Vysotski S. et al. | 2018 | USA | 206 | 61,2 | 20-84 | 53,1 | 171 | 8 |
| Wongsripuemtet J. et al. | 2018 | USA | 66 | 57,6 | 18-75 | 40,8 | 62 | 7 |
| Lemée J.M. et al. | 2019 | FRA | 50 | 68 | 18-75 | 49,6 | 42 | 8 |
| Liouta E. et al. | 2019 | GR | 69 | 46,4 | 18-78 | 50 | 49 | 6 |
| Metwahli H. et al. | 2019 | GEO | 30 | 53,3 | 18-77 | 54 | 30 | 6 |
| Kumar V.A. et al. | 2020 | USA | 134 | 57,1 | 17-78 | 47,5 | 43 | 6 |
| Rigolo L. et al. | 2020 | USA | 71 | - | - | - | - | 7 |
| Weiss Lucas C. et al. | 2020 | DE | 36 | 58 | - | 56 | 26 | 9 |
| Voets N.L. et al. | 2021 | UK | 71 | 56,3 | 19-70 | 41,9 | 69 | 8 |
| Muir M. et al. | 2022 | USA | 28 | 53,6 | 25-71 | 52 | 28 | 8 |

**Supplementary materials - Table 2.** Extended table showing the list of all the included studies (fMRI) and their numeric features.

| Author | Year | Country | N. of patients | Sex  distribution (% M) | Age range | Mean age | N. of gliomas | JBI |
| --- | --- | --- | --- | --- | --- | --- | --- | --- |
| Picht T. et al. | 2009 | DE | 10 | 50 | 42-74 | 58,9 | 6 | 7 |
| Picht T. et al. | 2011 | DE | 20 | 55 | 25-80 | 59 | 8 | 8 |
| Krieg S. et al. | 2012 | DE | 26 | 50 | 18-78 | 57,6 | 18 | 7 |
| Krieg S. et al. | 2012 | DE | 31 | 51,6 | 18-78 | 55,8 | 21 | 7 |
| Picht T. et al. | 2012 | DE | 73 | 64,4 | 46-66 | 57 | 33 | 7 |
| Picht T. et al. | 2013 | DE | 20 | 45 | - | 48 | 18 | 8 |
| Picht t. et al. | 2013 | DE | 11 | 72,7 | 20-59 | 38 | 11 | 8 |
| Tarapore P.E. et al. | 2013 | USA | 12 | 58,3 | 29-65 | 45 | 10 | 7 |
| Zdunczyk A. et al. | 2013 | DE | 10 | 60 | 24-49 | 35 | 3 | 7 |
| Conti A. et al. | 2014 | ITA | 20 | 40 | 19-76 | 51,4 | 14 | 7 |
| Frey D. et al. | 2014 | DE | 250 | 55,6 | 19-82 | 54 | 128 | 9 |
| Krieg S. et al. | 2014 | DE | 100 | 59 | - | 53,1 | 66 | 8 |
| Rizzo V. et al. | 2014 | ITA | 17 | 41,2 | 26-82 | 51,1 | 9 | 8 |
| Ille S. et al. | 2015 | DE | 35 | 62,9 | 24-74 | 44,3 | 30 | 9 |
| Krieg S. et al. | 2015 | DE | 140 | 64,3 | - | 59,2 | 140 | 9 |
| Picht T. et al. | 2015 | DE | 127 | 54,3 | 20-79 | 53,7 | 127 | 10 |
| Rosenstock T. et al. | 2016 | DE | 113 | 51 | 20-82 | 51 | 113 | 7 |
| Sollmann N. et al. | 2016 | DE | 100 | 57 | 19-84 | 53,5 | 62 | 7 |
| Sollmann N. et al. | 2016 | DE | 37 | 62,2 | 20-66 | 40 | 32 | 7 |
| Moser T. et al. | 2017 | DE | 43 | 55,8 | 24-78 | 54,4 | 43 | 7 |
| Raffa G. et al. | 2017 | ITA | 16 | 62,5 | 28-71 | 50,2 | 16 | 8 |
| Rosenstock T. et al. | 2017 | DE | 30 | 67 | - | 55 | 30 | 8 |
| Schwarzer V. et al. | 2017 | DE | 101 | 55,4 | - | - | 84 | 7 |
| Sollmann N. et al. | 2017 | DE | 100 | 57 | 19-84 | 54,1 | 52 | 7 |
| Sollmann N. et al. | 2017 | DE | 86 | 57 | - | 52,7 | 69 | 8 |
| Sollmann N. et al. | 2017 | DE | 40 | 62,5 | 19-80 | 53,8 | 27 | 8 |
| Takahura T. et al. | 2017 | JAP | 14 | 57,1 | - | 39 | 14 | 8 |
| Butenschön V.M. et al. | 2018 | DE | 1000 | 64,3 | - | 58 | 1000 | 6 |
| Jung J. et al. | 2018 | UK | 35 | 48,6 | 19-70 | 47 | 31 | 7 |
| Raffa G. et al. | 2018 | ITA | 105 | - | - | - | 75 | 9 |
| Raffa G. et al. | 2018 | ITA | 20 | 70 | 38-77 | 58,3 | 17 | 7 |
| Sollmann N. et al. | 2018 | DE | 60 | 63,3 | 23-73 | 47,6 | 60 | 8 |
| Lavrador J.P. et al. | 2019 | UK | 10 | 60 | 30-68 | 61,5 | 3 | 6 |
| Raffa G. et al. | 2019 | ITA | 41 | 65,9 | - | 59,3 | 41 | 8 |
| Sollmann et al. | 2019 | DE | 50 | - | - | - | 50 | 6 |
| Bährend I. et al. | 2020 | DE | 25 | 56 | 28-78 | 48 | 24 | 6 |
| Motomura K. et al. | 2020 | JAP | 61 | 63,9 | 18-72 | 41,1 | 58 | 8 |
| Sollmann N. et al. | 2020 | DE | 216 | 58,8 | 18-89 | 57 | 189 | 8 |
| Sollmann N. et al. | 2020 | DE | 34 | - | - | 56 | 18 | 7 |
| Zhang H. et al. | 2020 | DE | 39 | 69,2 | 25-79 | 56,3 | 24 | 8 |
| Zhang H. et al. | 2020 | DE | 20 | 80 | 52-80 | 63,2 | 20 | 9 |
| Belotti F. et al. | 2021 | DE | 183 | 62 | 21-81 | 50 | 183 | 9 |
| Hendrix P. et al. | 2021 | DE | 105 | 56,2 | - | 62,5 | 52 | 7 |
| Raffa G. et al. | 2021 | DE | 20 | 50 | 25-78 | 58,4 | 15 | 8 |
| Ille S. et al. | 2021 | DE | 147 | 53,8 | 20-84 | 54 |  | 8 |
| Rosenstock T. et al. | 2021 | DE | 66 | 59,1 | - | 48 | 66 | 7 |
| Rosenstock T. et al. | 2021 | DE | 165 | 64,8 | - | 50 | 165 | 9 |

**Supplementary materials - Table 3.** Extended table showing the list of all the included studies (nTMS) and their numeric features.
